# A biomarker study in long-lasting amnestic mild cognitive impairment

**DOI:** 10.1186/s13195-018-0369-8

**Published:** 2018-04-25

**Authors:** Chiara Cerami, Alessandra Dodich, Sandro Iannaccone, Giuseppe Magnani, Roberto Santangelo, Luca Presotto, Alessandra Marcone, Luigi Gianolli, Stefano F. Cappa, Daniela Perani

**Affiliations:** 1 Division of Neuroscience, San Raffaele Scientific Institute, Milan, Italy; 2 Clinical Neuroscience Department, San Raffaele Turro Hospital, Milan, Italy; 3 Neurology Department, San Raffaele Hospital, Milan, Italy; 4 Nuclear Medicine Department, San Raffaele Hospital, Milan, Italy; 5 NeTS Center, Istituto Universitario di Studi Superiori, Pavia, Italy; 6 IRCCS S. Giovanni di Dio Fatebenefratelli, Brescia, Italy; 7 Università Vita-Salute San Raffaele, Milan, Italy

**Keywords:** Positron emission tomography, Mild cognitive impairment, Alzheimer’s disease, Medial temporal lobe dysfunction, FDG-PET, Amyloid-PET

## Abstract

**Background:**

Mild cognitive impairment (MCI) is a heterogeneous syndrome resulting from Alzheimer’s disease (AD) as well as to non-AD and non-neurodegenerative conditions. A subset of patients with amnestic MCI (aMCI) present with an unusually long-lasting course, a slow rate of clinical neuropsychological progression, and evidence of focal involvement of medial temporal lobe structures. In the present study, we explored positron emission tomography (PET) and cerebrospinal fluid (CSF) biomarkers in a sample of subjects with aMCI with such clinical features in order to provide in vivo evidence to improve disease characterisation in this subgroup.

**Methods:**

Thirty consecutive subjects with aMCI who had long-lasting memory impairment (more than 4 years from symptom onset) and a very slow rate of cognitive progression were included. All subjects underwent fluorodeoxyglucose-positron emission tomography (FDG-PET) metabolic imaging. A measure of cerebral amyloid load, by PET and/or CSF, was obtained in 26 of 30 subjects. The mean clinical follow-up was 58.3 ± 10.1 months.

**Results:**

No patient progressed to dementia during the follow-up. The typical AD FDG-PET pattern of temporoparietal hypometabolism was not present in any of the subjects. In contrast, a selective medial temporal lobe hypometabolism was present in all subjects, with an extension to frontolimbic regions in some subjects. PET imaging showed absent or low amyloid load in the majority of samples. The values were well below those reported in prodromal AD, and they were slightly elevated in only two subjects, consistent with the CSF β-amyloid (1–42) protein values. Notably, no amyloid load was present in the hippocampal structures.

**Conclusions:**

FDG-PET and amyloid-PET together with CSF findings questioned AD pathology as a unique neuropathological substrate in this aMCI subgroup with long-lasting disease course. The possibility of alternative pathological conditions, such as argyrophilic grain disease, primary age-related tauopathy or age-related TDP-43 proteinopathy, known to spread throughout the medial temporal lobe and limbic system structures should be considered in these patients with MCI.

## Background

Mild cognitive impairment (MCI) is a heterogeneous syndrome that can be due to Alzheimer’s disease (AD) and non-AD pathologies [1]. The presence of an early and significant objective deficit of episodic memory is considered the main criterion supporting the diagnosis of typical AD and the best cognitive predictor of the development of AD dementia [2, 3]. Though the amnestic syndrome of hippocampal type is the most typical presentation in prodromal AD, impairments in delayed recall tasks may be present in individuals with non-AD disorders, such as the behavioural variant of frontotemporal dementia (bvFTD) [4], argyrophilic grain disease (AGD) [5, 6] and the recently identified suspected non-AD pathology (SNAP) [7–9].

Subjects with selective long-term memory impairment and a relatively stable or very slowly progressing (up to decades) condition have been reported [10, 11]. This condition has been considered up to now as a phenotypical expression of a focal medial temporal lobe dysfunction possibly due to AD pathology [10–12]. In particular, patients with AD with a limbic predominant deposition of tau protein present with a specific phenotype (e.g., old age at onset, predominant amnestic syndrome) and selective medial temporal lobe atrophy visualised by magnetic resonance imaging (MRI) compared with the other AD variants (i.e., typical AD and hippocampus-sparing AD) [13].

Recently, patients with MCI with such a clinical phenotype and no clear in vivo evidence of AD pathology were reported in the literature as part of the heterogeneous clinical group labelled SNAP [8, 9, 14–17]. This is a biomarker-based concept applied to individuals with evidence of neurodegeneration in the absence of cerebral amyloid load [9]. This definition reflects the notion that pathologies other than AD may underlie neurodegenerative changes, as revealed by cerebrospinal fluid (CSF) and fluorodeoxyglucose-positron emission tomography (FDG-PET) biomarkers, in subgroups of patients clinically presenting with an AD-like phenotype.

In the present study, we assessed FDG-PET brain hypometabolism and cerebral amyloid load by PET imaging as well as CSF β-amyloid (1–42) protein (Aβ_42_) and tau values in a sample of subjects with MCI with predominant episodic memory impairment and a very slow rate of progression. The aim was to provide in vivo evidence to improve disease characterisation in this MCI subgroup.

## Methods

### Participants

The sample included 30 subjects (mean age 74.1 ± 4.8 years; mean education level 10.3 ± 4.5 years; mean disease duration at first evaluation 44.5 ± 25.5 months) fulfilling the Petersen criteria [1] for amnestic MCI (aMCI) and characterised by (1) a predominant episodic memory impairment, (2) a long-term clinical course (i.e., more than 4 years) and (3) a slow rate of progression of memory deficits. All subjects were consecutively referred to the neurology centres of San Raffaele Hospital (Milan, Italy). All of the included patients had a 3 to 5 years of clinical follow-up (i.e., 58.3 ± 10.1 months).

Conventional MRI was used to exclude the presence of white matter hyperintensities and lacunes of cerebrovascular origin as causes of cognitive impairments. Upon conventional inspection of MRI scans, hippocampal atrophy was found in the majority of subjects, without a clear radiological picture of hippocampal sclerosis (i.e., reduction of hippocampal volume with abnormal shape of mesial temporal lobe structures observed on T1-weighted images and increased signal intensity on T2-weighted and fluid-attenuated inversion recovery images) [18]. All of the included subjects had an FDG-PET scan, and 26 of 30 subjects with MCI had an amyloid biomarker evaluation (i.e., either with amyloid-PET and/or CSF Aβ_42_ measure). *See* Tables 1 and 2 for details.

**Table 1:** Demographic and clinical features of the sample

	Patient sample (*n* = 30)
Female/male ratio	12/18
Age in years (mean ± SD)	74.1 ± 4.8
Age range in years	65–84
Years of education (mean ± SD)	10.3 ± 4.5
Disease duration in months at first evaluation (mean ± SD)	44.5 ± 25.5
Months of follow-up (mean ± SD)	58.3 ± 10.1
Disease duration in months at follow-up (mean ± SD)	102.8 ± 28.1
MMSE adjusted score at first evaluation (mean ± SD)	26.5 ± 2.1
MMSE adjusted score at follow-up (mean ± SD)	25.1 ± 2.5
CDR Sum of Boxes at first evaluation (mean ± SD)	1.91 ± 0.6
CDR Sum of Boxes at follow-up (mean ± SD)	2.9 ± 1.7
Diagnosis at first evaluation	15 s-aMCI, 15 m-aMCI
Diagnosis at follow-up	9 s-aMCI, 21 m-aMCI
Lumbar puncture (no. of subjects)	20/30
CSF Aβ_42_ and t-tau/p-tau normal values	7/20
CSF Aβ_42_ low values	10/20
CSF p-Tau/t-tau high values	3/20
Amyloid-PET (no. of subjects)	16/30
SUVr values from 1.0 to 1.45	8/16
SUVr values from 1.45 to 1.80	6/16
SUVr values from 1.80 to 1.90	2/16

*Abbreviations: MMSE* Mini Mental State Examination, *CDR* Clinical Dementia Rating, *CSF* Cerebrospinal fluid, *Aβ*_*42*_ β-Amyloid (1–42) protein, *t-tau* Total tau, *p-tau* Phosphorylated tau, *s-aMCI* Single-domain amnestic mild cognitive impairment, *m-aMCI* Multiple-domain amnestic mild cognitive impairment, *PET* Positron emission tomography

**Table 2:** Demographic data, clinical features and biomarker findings in each enrolled patient. The table shows patients’ findings according to the biomarkers (n. 26 with FDG-PET data and amyloid measures, either by amyloid-PET study or CSF assessment; n.4 with FDG-PET data)

	Gender	Age	Education	MMSE at the first evaluation	Diagnosis at the first evaluation	Disease duration at the first evaluation	MMSE at the follow-up	Diagnosis at the follow-up	Disease duration at the follow-up	FDG-PET pattern	Amyloid-PET neocortical SUVr values	CSF Aβ42	CSF t-Tau	CSF p-Tau	t-Tau/ Aβ42 Ratio
#1	F	79	17	26	s-aMCI	26	24	s-aMCI	81	C	1.38	752	222	45	0.29
#2	M	77	6	28	s-aMCI	48	27	s-aMCI	145	C	1.16	957	243	35	0.25
#3	M	71	13	29	m-aMCI	108	28	m-aMCI	163	B	1.15	630	122	30	0.19
#4	M	83	17	24	m-aMCI	36	22	m-aMCI	118	C	1.09	1000	505	71	0.50
#5	M	75	5	23	m-aMCI	60	25	m-aMCI	119	C	1.18	720	174	35	0.24
#6	M	72	13	27	s-aMCI	72	28	s-aMCI	127	B	1.63	255	352	82	1.38
#7	M	79	18	25	s-aMCI	72	23	m-aMCI	115	C	1.90	252	293	73	1.16
#8	M	68	8	29	s-aMCI	36	25	m-aMCI	91	B	1.44	400	275	52	0.68
#9	M	70	5	25	s-aMCI	48	26	m-aMCI	115	B	1.21	411	638	102	1.55
#10	M	84	13	28	m-aMCI	96	22	m-aMCI	151	C	1.59	596	171	39	0.28
#11	M	77	11	25	s-aMCI	48	25	s-aMCI	103	B	1.66	-	-	-	-
#12	M	69	8	27	s-aMCI	36	26	s-aMCI	83	B	1.69	-	-	-	-
#13	F	71	12	24	s-aMCI	24	25	s-aMCI	79	C	1.83	-	-	-	-
#14	F	69	8	29	m-aMCI	24	22	m-aMCI	79	C	1.71	-	-	-	-
#15	M	77	3	24	m-aMCI	36	23	m-aMCI	91	C	1.19	-	-	-	-
#16	F	76	5	27	m-aMCI	60	26	m-aMCI	115	C	1.59	-	-	-	-
#17	M	71	18	28	s-aMCI	24	28	s-aMCI	79	A	-	742	259	42	0.34
#18	F	69	17	27	s-aMCI	108	26	m-aMCI	175	A	-	270	339	70	1.25
#19	F	65	8	28	s-aMCI	24	27	m-aMCI	85	B	-	497	489	105	0.98
#20	F	68	5	25	m-aMCI	60	23	m-aMCI	118	B	-	365	343	59	0.94
#21	M	76	8	27	m-aMCI	18	26	m-aMCI	73	B	-	734	268	45	0.36
#22	F	80	10	26	m-aMCI	24	21	m-aMCI	91	A	-	355	763	108	2.15
#23	M	78	13	29	m-aMCI	24	27	m-aMCI	73	C	-	1010	379	87	0.37
#24	M	71	8	28	m-aMCI	24	26	m-aMCI	79	B	-	308	886	134	2.87
#25	M	77	13	29	m-aMCI	60	28	m-aMCI	123	C	-	847	379	84	0.45
#26	F	75	12	24	m-aMCI	24	23	m-aMCI	79	A	-	454	244	48	0.54
#27	F	73	5	25	s-aMCI	50	25	s-aMCI	79	C	-	-	-	-	-
#28	M	79	13	28	s-aMCI	120	27	s-aMCI	83	C	-	-	-	-	-
#29	F	69	13	29	s-aMCI	60	27	m-aMCI	87	C	-	-	-	-	-
#30	F	76	5	21	m-aMCI	52	21	m-aMCI	85	B	-	-	-	-	-

*MMSE* Mini Mental State Examination; *CSF* cerebrospinal fluid; *Aβ42* β Amyloid (1-42) protein; *t-Tau* total tau; *p-Tau* phosphorylated tau; *M* male; *F* female; *s-aMCI* single-domain amnestic Mild Cognitive Impairment; *m-aMCI* multiple domain amnestic Mild Cognitive Impairment; *FDG-PET pattern A* selective hypometabolism of medial temporal lobe structures (hippocampus and/or hippocampal structures); *FDG-PET pattern B* hypometabolism of medial temporal lobe structures and posterior cingulate cortex; *FDG-PET pattern C* extensive hypometabolism involving medial temporal lobe structures and other fronto-limbic structures

### CSF measures

CSF was obtained from 20 of 30 subjects near the time of first clinical evaluation (< 3 months) by lumbar puncture in the L3-L4 or L4-L5 interspace. The procedure was performed early in the morning. No serious adverse events were reported. CSF (8–10 ml) was collected in sterile polypropylene tubes. Part of it was used to determine routine chemical parameters (leucocyte and erythrocyte cell count, glucose measurement, protein total content). The remaining CSF was centrifuged for 10 min at 4000 × *g* at 4 °C and stored at − 80 °C until analysis to ensure the stability of the CSF biomarkers. Measurement of CSF Aβ_42_, total tau (t-tau) and phosphorylated tau (p-tau) levels was performed using commercial available enzyme-linked immunosorbent assay (ELISA) kits according to the manufacturer’s protocol and blinded to clinical data. Normal values were set ≥ 500 ng/L for Aβ_42_ values, ≤ 450 ng/L (if age was 51–70 years) or < 500 ng/L (if age was > 71 years) for t-tau values and ≤ 61 ng/L for p-tau values, according to the ELISA kit guidelines and literature recommendations [19].

### FDG-PET imaging

FDG-PET scans were acquired in 30 of 30 patients near the time of first clinical evaluation (< 3 months) according to European Association of Nuclear Medicine guidelines [20]. FDG-PET was performed in each subject at the Nuclear Medicine Unit, San Raffaele Hospital (Milan, Italy), with the Discovery STE multi-ring PET-computed tomography (CT) system (GE Medical Systems, Milwaukee, WI, USA). Before radiopharmaceutical injection of FDG (usually 185–250 MBq via a venous cannula), subjects were fasted for at least 6 h, and the measured blood glucose threshold was < 120 mg/dl. All images were acquired with an interval between injection and scan start of 45 min and scan duration of 15 min. Images were reconstructed using an ordered subset expectation maximisation (OSEM) algorithm. Attenuation correction was based on CT scans. Specific software integrated in the scanner was used for scatter correction. All subjects gave written informed consent after detailed explanation of the FDG-PET procedure.

Image pre-processing was performed using statistical parametric mapping (SPM; http://www.fil.ion.ucl.ac.uk/spm/software) according to an optimised SPM procedure with implementation of a standardised SPM FDG dementia-specific template [21] for spatial normalisation. This is an optimised method validated in MCI and different dementia conditions at the single-subject level and showing high accuracy in estimating specific metabolic patterns [22–24]. In detail, images were smoothed with an 8-mm FWHM gaussian kernel; proportional scaling was used to remove inter-subject global variation in PET intensity; and each FDG-PET scan was then tested for relative “hypometabolism” by means of a two-samples *t* test implemented in SPM in a comparison with a normal FDG-PET image database (*n* = 112) on a voxel-by-voxel basis, with age as a covariate [22]. The FDG-PET image database included data acquired with different PET scanners. However, this did not affect data analysis with the optimised SPM procedure as evaluated and reported by Presotto et al. [25] with a large sample of FDG-PET images acquired with several PET scanners. The statistical threshold for the analysis at single-subject level was set at *p* = 0.05, familywise error (FWE)-corrected for multiple comparisons at the voxel level. Only clusters containing more than 100 voxels (i.e., 800 mm^3^) were deemed to be significant.

In order to compute brain metabolic changes at the group level, we also performed a second-level SPM analysis (one-sample *t* test) in the whole MCI group. The threshold was set at *p* < 0.05, FWE-corrected for multiple comparisons at the voxel level. In order to reveal more subtle effects in brain hypometabolism, a *p* < 0.001 uncorrected analysis (FWE-corrected at the cluster level) was also performed.

### Amyloid-PET imaging

Amyloid-PET imaging was performed in 16 of 30 subjects using the [^18^F]florbetaben NeuraCeq™ tracer (Piramal Imaging, Berlin, Germany). All amyloid-PET acquisitions were performed within a mean of 3–12 months (i.e., 5.6 ± 1.9 months) from the first clinical evaluation. Patients received a single intravenous bolus injection of 315.6 ± 16.6 MBq of tracer. PET scans were acquired using a hybrid GE Discovery PET/CT 690 system (interval between injection and scan start 90 min; scan duration 15 min) [26]. CT scans were used for attenuation correction of PET data. All image reconstruction was performed using a 3D-OSEM algorithm. Images were scaled by division through the respective median cerebellar grey matter voxel intensity, and image processing resulted in regional cortical grey matter tracer uptake values relative to cerebellar grey matter (i.e., standardised uptake value [SUV]). SUVs were calculated for all regions according to the procedure reported by Villemagne et al. [27]. In particular, we considered ROIs in frontal (i.e., dorsolateral, ventrolateral and orbitofrontal regions), superior and inferior parietal, lateral temporal, lateral occipital, and anterior and posterior cingulate cortices. Each SUV was used to derive the composite SUV ratio (SUVr) referenced to the cerebellar cortex, a region most frequently used in SUV analysis because it is relatively unaffected by Aβ plaques in sporadic AD [27]. In addition to the above-mentioned cortical regions suggested in the literature to calculate the composite neocortical SUVr, we selected ROIs in hippocampal structures, amygdala and insula according to the Automated Anatomical Labelling atlas. This further analysis was done in order to test in vivo with PET whether amyloid deposits leading to a major effect on neurodegeneration in these brain regions might selectively exist. The long-lasting disease duration in subjects with MCI with selective long-term memory impairment or relatively stable or very slow progression has indeed been attributed to focal medial temporal lobe dysfunction possibly due to AD pathology [10–12]. Finally, a non-parametric correlation analysis (Spearman’s rho) was performed to evaluate the relationship between mean neocortical amyloid-PET SUVr values and CSF Aβ_42_ values in those patients who underwent both amyloid-PET and lumbar puncture.

## Results

### Clinical neuropsychological findings

At the time of the first clinical evaluation, the subjects’ age ranged from 65 to 84 years, and both sexes were similarly affected. Fifteen subjects were classified as having single-domain aMCI (s-aMCI), and 15 were classified as having multiple-domain aMCI (m-aMCI). Of note, 21 of 30 subjects showed behavioural disturbances (i.e., anxiety, irritability, aggressiveness, apathy and emotional blunting). At the clinical follow-up (58.3 ± 10.1 months), none had progressed to dementia. The majority of subjects with aMCI showed stable profiles (i.e., 9 with s-aMCI and 15 with m-aMCI). Mild progression of memory impairment and additional cognitive deficits were found in 6 subjects with s-aMCI, who were reclassified as having m-aMCI. *See* Tables 1 and 2 for demographic and clinical details.

Subjects with MCI who had some evidence of amyloid deposition (*n* = 15) visualised by CSF or PET imaging (*see below*) did not show any significant difference in clinical or neuropsychological features compared with amyloid-negative (*n* = 11) subjects, except for the female/male ratio and the disease duration in months, with amyloid-negative subjects showing longer disease duration (Table 3).

**Table 3:** Clinical and neuropsychological features of the MCI sample grouped according to the evidence (i.e., Aβ+ MCI) or the absence (i.e., Aβ- MCI) of amyloid pathology by amyloid-PET and/or CSF imaging. Amyloid pathology information was available in 26/30 MCI subjects. Values are shown as mean ± standard deviation

	Aβ+ MCI n=15	Aβ- MCI n=11	Statistics
Female/Male ratio	8/7	11/1	p<0.05
Age in years	73.5±5.4	75.3±4.5	p=0.4
Years of education	9.2±4.8	11.1±4.9	p=0.3
Disease duration in months	30.3±16	50.3±29	p<0.05
Diagnosis at the first evaluation			
Mini Mental State Examination (cut-off = 24)	25±3.9	26.5±2.9	p= 0.31
Token test (cut-off = 26.25)	30.8±4.3	30.7±2	p=0.9
Phonemic fluency (cut-off = 16)	24.1±12.2	20±7.4	p=0.34
Semantic fluency (cut-off = 24)	28.8±10.5	27.1±5.7	p=0.64
Digit Span (cut-off = 3.5)	5.23±1.3	5.5±1.1	p=0.66
Corsi Span (cut-off = 3.5)	4.7±0.8	4.3±0.7	p=0.25
Rey Auditory Verbal Learning Test immediate recall (cut-off = 28.5)	19.17±3.7	20.3±5.1	p=0.55
Rey Auditory Verbal Learning Test delayed recall (cut-off = 4.68)	1.42±1.9	1.22±1.5	p=0.80
Rey-Osterrieth Complex Figure recall (cut-off = 9.46)	6.25±3.1	6.25±5.2	p=1
Rey-Osterrieth Complex Figure copy (cut-off = 28.87)	22.6±9	28.64±6.1	p=0.075
Attentive Matrices (cut-off = 30)	43.85±7	45.18±10.7	p=0.72
Raven Matrices (cut-off = 17.5)	24.9±7.1	22.73±6.1	p=0.52

### CSF findings

Among the 20 subjects with CSF data available, 7 had completely normal CSF values. Three subjects showed only slight elevation of p-tau levels, suggesting the presence of a neurodegenerative process. The remaining ten subjects showed Aβ values below the cut-off proposed for Aβ positivity [19]. Half of these subjects (i.e., five of ten) had only slightly altered Aβ results, with normal t-tau and p-tau values found in three of them. In these latter three subjects, MCI due to AD had a low probability according to the Erlangen Score Algorithm for the interpretation of CSF results [28, 29]. Pathological Aβ results, with slight alteration of p-tau levels, were present in three subjects. Only two subjects showed clearly pathological results for both Aβ and t-tau/p-tau, compatible with an AD-like CSF profile [29]. *See* Table 2 for further details on individual-subject values. The t-tau/Aβ ratio confirmed an AD-like profile in those subjects with clearly pathological Aβ values. Ratios above the cut-off proposed for AD positivity as reported by Shaw et al. [30] were also found in four of the subjects with an unclear single-biomarker profile. *See* Table 2 for details on individual-subject values.

### FDG-PET imaging findings

**Fig. 1:**
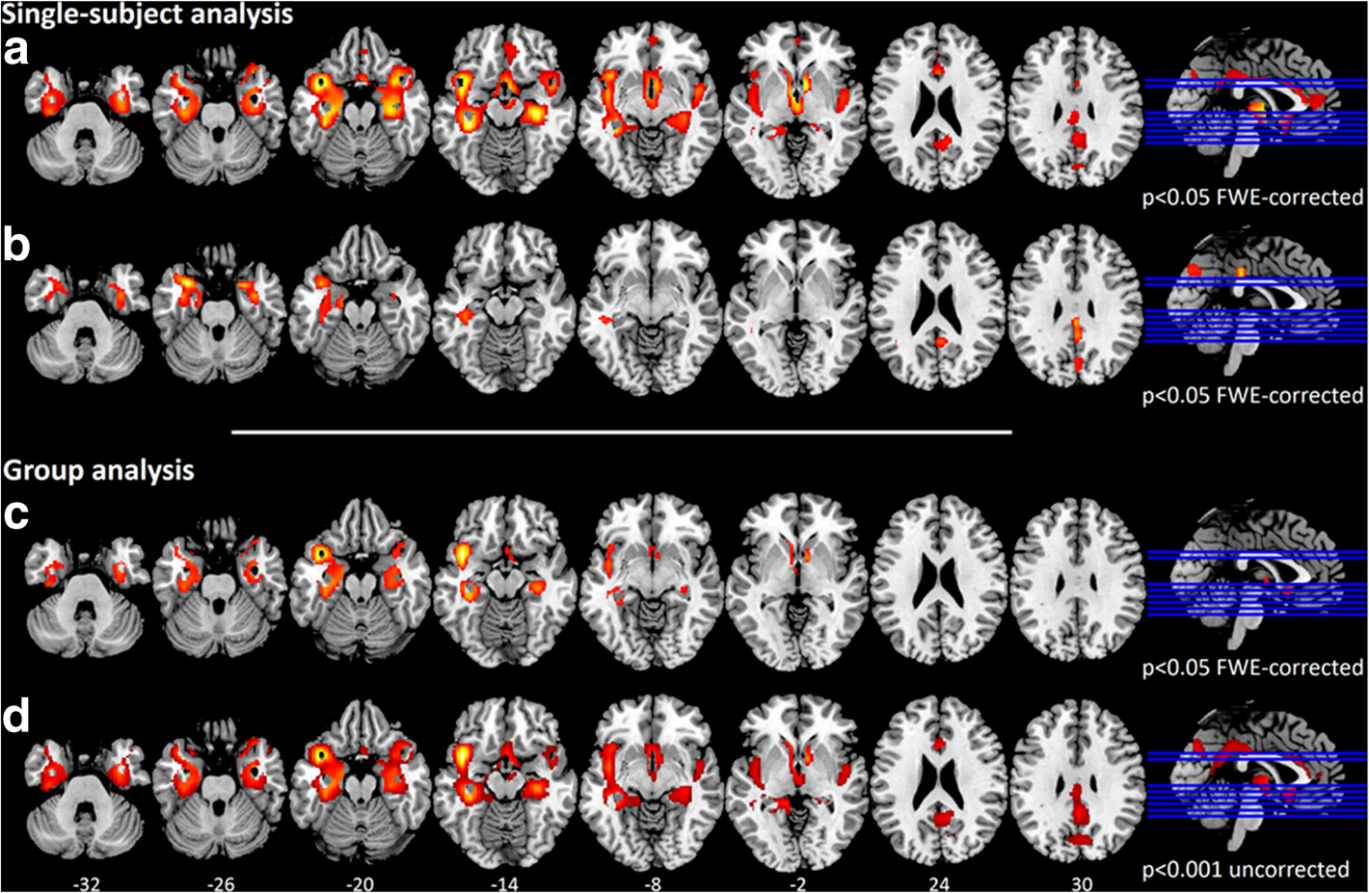
**a** and **b** Fluorodeoxyglucose-positron emission tomography (FDG-PET) hypometabolic patterns of two example subjects (i.e., statistical parametric mapping [SPM] single-subject analysis: 1 patient vs. 112 control subjects; *p* < 0.05, familywise error [FWE]-corrected at the voxel level with *k* > 100 voxels) representing (**a**) extensive hypometabolism in the medial temporal lobe (i.e., hippocampus and hippocampal structures) and limbic structures (amygdala, frontomedial cortex, and insula) and (**b**) selective medial temporal lobe hypometabolism, more extended on the left side. **c** and **d** FDG-PET hypometabolic pattern of the whole patient group (i.e., SPM one-sample group analysis; *p* < 0.05 FWE-corrected at the voxel level and *p* < 0.001 uncorrected at the voxel level, FWE-corrected at cluster level)

No subject showed the typical AD temporoparietal hypometabolism on the basis of FDG-PET imaging [22–24]. We found instead a consistent pattern of focal hypometabolism involving the hippocampus and/or hippocampal structures in every case. In half of the sample, additional metabolic changes were present in the frontomedial cortex, the amygdala, the posterior part of the insula extending to the parietal operculum and the superior temporal gyrus. Some patients (i.e., 11 of 30) showed posterior cingulate cortex hypometabolism. *See* Fig. 1a and b and Table 2 for details.

The group analysis of FDG-PET data using the SPM procedure showed a bilateral pattern of hypometabolism involving the hippocampal structures spreading to the insula on the left side (*p* < 0.05, FWE-corrected for multiple comparisons at the cluster level) (Fig. 1c). At the less stringent statistical threshold of significance (i.e., *p* < 0.001 uncorrected, FWE-corrected at the cluster level), we found a more extended hypometabolism only in the medial temporal lobe structures, without any involvement of the lateral temporoparietal regions (Fig. 1d).

### Amyloid-PET imaging findings

**Fig. 2:**
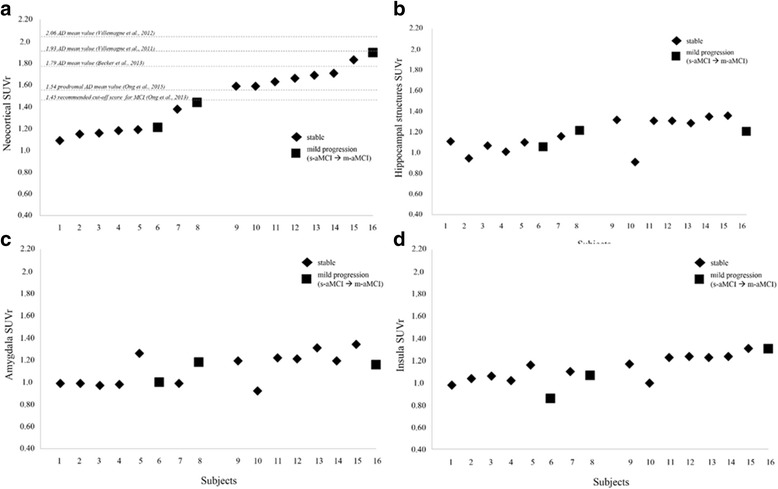
Distribution of the mean neocortical standardized uptake value ratio (SUVr) values (**a**) and SUV values in the hippocampal structures (**b**), amygdala (**c**) and insula (**d**) in those patients who underwent amyloid-positron emission tomographic imaging. Reference thresholds for amyloid positivity of the composite SUVr values in patients with mild cognitive impairment (MCI) and patients with Alzheimer’s dementia (AD) are reported. *m-aMCI* Multiple-domain amnestic mild cognitive impairment, *s-aMCI* Single-domain amnestic mild cognitive impairment

Negative or very low levels of tracer uptake were found in the majority of subjects with MCI (Table 2). In particular, 8 of 16 patients had very low composite SUVr values (1.22 ± 0.12), comparable to those reported in the literature for Aβ*-*negative healthy control subjects [27, 31–35], and notably well below the proposed cut-off for prodromal AD (i.e., < 1.45) [33]. Low to intermediate SUVr values (1.45 < SUVr < 1.80) below the mean values reported in the literature for AD Aβ positivity were found in 6 of 16 subjects (*see* Fig. 2). Only 2 of 16 subjects had slightly elevated composite SUVr values (i.e., 1.83 and 1.90) (Fig. 2). No regional effect of the tracer retention in the single cortical ROIs included in the composite SUVr measure emerged. Notably, in all subjects with MCI, there was a sparing of the hippocampal structures, amygdala and insula, in which SUVr values were always low overall (< 1.4). *See* Fig. 2 for details.

### CSF Aβ_42_ and amyloid-PET imaging correspondence

**Fig. 3:**
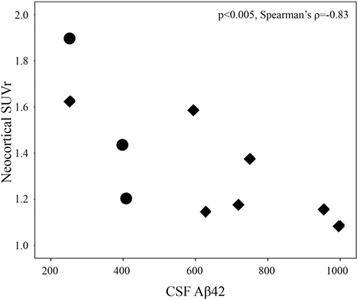
Scatterplot showing the inverse correlation between mean neocortical standardized uptake value ratio (SUVr) and cerebrospinal fluid (CSF) β-amyloid (1–42) protein (Aβ_42_) values in those subjects who underwent both amyloid-positron emission tomography and lumbar puncture

Amyloid-PET SUVr and CSF Aβ_42_ values were consistent (Fig. 3). In detail, seven subjects had normal or slightly reduced CSF Aβ_42_ values and no amyloid-PET burden, one had a normal CSF Aβ_42_ value but a slight increase in SUVr value, and two had reduced CSF Aβ_42_ levels and increased SUVr values. There was a significant negative correlation between mean neocortical SUVr and CSF Aβ_42_ values (Spearman’s rho = − 0.83, *p* < 0.005).

## Discussion

Our sample of subjects with aMCI with prevalent or exclusive long-term memory deficits and a slow cognitive progression did not show the FDG-PET hypometabolism pattern typical of AD. They had reduced glucose metabolism in the medial temporal lobe structures with no amyloid load visualised by PET imaging in these structures (*see* Figs. 1 and 2).

In addition to the consistent pattern of reduced metabolism in the hippocampal structures, hypometabolism in the frontomedial cortex, insula and anterior superior temporal cortex was present in some subjects. A few subjects showed reduction of glucose metabolism in the posterior cingulate cortex, which can be interpreted as a functional disconnection effect due to the severe involvement of the hippocampal structures. As reported in the literature, even in the absence of grey matter loss or amyloid toxicity in the posterior cingulate cortex, grey matter loss in the medial temporal lobe structures is sufficient to cause remote metabolic effects in connected regions [36].

This FDG-PET hypometabolic pattern involving limbic structures is similar to the pattern reported in some patients with bvFTD [37, 38]. The subjects in our series, however, presented with a clinical phenotype of aMCI, and the additional mild behavioural changes found in many subjects did not fulfil the clinical criteria for bvFTD [39] at either onset or follow-up.

As for the amyloid load visualised by [^18^F]florbetaben-PET or CSF, we found evidence of β-amyloidosis in some subjects according to the amyloid-PET SUVr (i.e., 8 of 16 subjects showed intermediate or high SUVr values) and/or CSF Aβ_42_ cut-off values [19] (i.e., 10 of 20 showed low CSF Aβ_42_ values). Notably, the cognitive profile and FDG-PET features of subjects with evidence of in vivo β-amyloidosis did not show differences from patients with MCI without amyloid load (*see* Fig. 2 and Table 3). In addition, they did not progress to dementia during long-term follow-up (i.e., 58.3 ± 10.1 months). The clinical value of β-amyloidosis as a marker of prodromal AD in these subjects is poor, however. It must be taken into consideration that a consistent percentage of cognitively normal subjects (i.e., 16–30% [27, 32]), as well as non-AD neurodegenerative patients [27], show incidental amyloid load with mean neocortical SUVr values above the proposed cut-off score for prodromal AD [40]. This is particularly true in elderly populations such as the one included in our study [32, 40]. Thus, the β-amyloidosis seen in these subjects does not per se provide conclusive evidence for an “MCI due to AD” condition.

A pure or mixed limbic-predominant AD variant [12] could be the possible underlying neuropathological substrate in those subjects presenting with low cortical amyloid uptake. However, it is unlikely in patients with no evidence of brain amyloidosis. The possibility of other pathological substrates needs to be considered in the presence of long-lasting aMCI. These include AGD, a late-onset tauopathy typically characterised by tau lesions spreading throughout the limbic system [5, 6, 41]. AGD usually affects elderly subjects (mean age of onset 75–80 years), without sex differences, and is clinically characterised either by a severe amnestic syndrome with relative sparing of other cognitive functions [5, 6] or by a clinical neuropsychological phenotype overlapping with that of typical AD dementia.

Mixed neuropathological conditions have been suggested as a possible underlying substrate in patients with MCI with a stable clinical picture. Multiple co-morbid neuropathologies were reported previously in a large neuropathological study of individuals (*n* = 1337) followed longitudinally from normal or MCI status to death, showing that less than one-fourth of patients with MCI had “pure” AD at autopsy, whereas more than half of the stable patients with MCI had mixed AD pathology changes [42]. Brain arteriolosclerosis was also reported as a possible neuropathological substrate in this MCI case series [42].

No subject in our MCI sample progressed to dementia during the follow-up, notably not even the two patients who showed higher SUVr values. This finding also supports a non-AD condition overall in this subgroup and once more suggests a possible combined AGD and additional amyloid pathological substrate in those individuals with slightly elevated SUVr values. Some subjects with AGD indeed showed an associated AD-type pathology [6]. AGD and AD pathologies may act as additive factors, because subjects with combined AGD with mild to moderate AD-type pathologies were more frequently associated with dementia progression than with pure AGD pathology [5, 43].

We found mild behavioural disturbances in the majority of our subjects with aMCI. Although the presence of emotional blunting could suggest a diagnosis of a bvFTD condition, no other behavioural changes typical of bvFTD (e.g., disinhibition; impulsiveness; perseverative, stereotypic or compulsive/ritualistic behaviour; hyperorality; and dietary changes) were present. Abnormal behaviours such as those observed in our sample represent common initial symptoms in pathology-confirmed AGD [44].

Although asymmetry of brain volume in medial temporal regions has been described in study of patients with advanced AGD [45, 46], this anatomostructural aspect was not reported by the expert radiologists who evaluated conventional MRI scans in our series. This work was indeed performed in a clinical setting, and advanced MRI measures were not available. Future studies are needed to highlight the role of subtle and specific anatomical changes in the differential diagnosis of this MCI group.

Other possible pathological substrates also need to be considered, such as primary age-related tauopathy (PART) [47] and TDP-43 pathology [48]. PART is a tauopathy in the absence of β-amyloidosis in which medial temporal lobe structures are primarily involved and abnormally elevated CSF tau protein levels are often observed [47]. Age-related deposition of TDP-43 protein is a neuropathological condition recently described in elderly people with impairments in episodic memory and no pathological diagnosis of AD or frontotemporal lobar degeneration [48]. According to Nag et al. [48], there is an association between this proteinopathy and age-related hippocampal sclerosis (i.e., reduction of the volume of CA1 and subiculum). Hippocampal sclerosis has been suggested by Bien et al. [49] as the main cause of memory loss in patients with MCI with predominant episodic memory impairment, slow progression of neuropsychological deficits and preservation of other cognitive functions. The large majority of elderly people with hippocampal sclerosis included in the Nag et al. cohort [48] had TDP-43 pathology.

Both PART and age-related TDP-43 proteinopathy can represent possible pathological substrates of the heterogeneous clinical spectrum called SNAP [9]. Reports on subjects with SNAP MCI [7–9, 14–17], as well subjects with PART [47] and age-related TDP-43 [48], indeed show some parallelism with the clinical features of our aMCI sample: advanced age, very mild cognitive symptoms, neurodegeneration without amyloidosis, and low risk of clinical neuropsychological decline. Only clinical and neuroimaging studies with neuropathological verification will provide conclusive results about the array of pathologies responsible for the clinical picture reported in such subjects with long-lasting stable amnestic MCI.

## Conclusion

The main contribution of the present in vivo combined biomarker study (FDG-PET for neurodegeneration and amyloid-PET and CSF for pathology) is that additional nosographic classifications besides the limbic AD variant need to be considered in patients with MCI with a long-lasting disease course and slowly progressing or non-progressing cognitive decline.
